# Attitudes towards gambling in Finland: a cross-sectional population study

**DOI:** 10.1186/1471-2458-14-982

**Published:** 2014-09-20

**Authors:** Anne H Salonen, Sari Castrén, Susanna Raisamo, Jim Orford, Hannu Alho, Tuuli Lahti

**Affiliations:** Department of Mental Health and Substance Abuse Services, National Institute for Health and Welfare, P.O. Box 30, Helsinki, Finland; Department of Alcohol, Drugs and Addiction, National Institute for Health and Welfare, Helsinki, Finland; Clinical and Community Psychology, School of Psychology, University of Birmingham, Birmingham, England; Research Unit of Substance Abuse Medicine, University of Helsinki, Helsinki, Finland

**Keywords:** Attitudes, Cross-sectional, Gambling, Population study

## Abstract

**Background:**

Attitudes towards gambling influence gambling behaviour but also reflect the existing gambling policy in a society. However, studies examining general attitudes towards gambling at the population level are scarce. The first aim of this study was to investigate general attitudes of the Finnish population towards gambling. The second aim was to explore the association of socio-demographics, gambling behaviours, being a concerned significant other (CSO) of a problem gambler and perceived health and lifestyle with attitudes towards gambling among the Finnish population.

**Methods:**

A cross-sectional study was performed by structured telephone interview on a random sample of 15-74-year-old Finns between October 2011 and January 2012. The data (n = 4484) was weighted based on age, gender and region of residence. Attitudes towards gambling were measured with the eight-item version of the Attitude Towards Gambling Scale (ATGS-8). A factor analysis was performed to test the structure of the Finnish version of the ATGS-8. The data were analysed using one-way ANOVA test, *t*-test and multiple regression analysis.

**Results:**

On average, attitudes of Finns towards gambling were negative. The most significant factors associated with positive attitudes towards gambling were male gender, young age, 12 years or more education and net income more than 2000€, low score on gambling severity, being a non-CSO of a problem gambler and high alcohol consumption

**Conclusions:**

The association between young age, male gender, high net income and risky alcohol consumption, and favourable gambling attitudes was strong, and also reflects risky gambling behaviour. Experiencing gambling-related harms caused by one’s own or significant other’s excessive gambling seems to indicate unfavourable attitudes towards gambling.

## Background

During the last decades gambling has become more popular in most societies simultaneously with the growth of gambling opportunities [1, 2]. As excessive gambling can cause adverse consequences like economic, social and health problems affecting the gamblers, their families and the society as a whole [3], regulatory policies have been introduced in several jurisdictions to prevent and reduce gambling-related harms [4, 5].

National gambling policies together with the cultural norms of the society impact public attitudes towards gambling. Public attitudes towards gambling can be divided into attitudes towards gambling in general or attitudes towards a specified form of gambling [6]. On average, attitudes towards gambling appear to be negative [6–10]. An example from Alberta, Canada found that community attitudes toward legalised gambling were stating clearly that gambling and public policy are misaligned to the extent that gambling’s harms are thought to outweigh its benefits [9].

In 2001, Volberg claimed that although the overall gambling participation rate had increased in the United States (US), attitudes towards gambling have remained fairly constant and are likely to become more negative over time [11]. On the other hand, research findings from the 2007 and 2010 British Gambling Prevalence Surveys indicated that public attitudes towards gambling have slightly become more positive amongst both genders [6, 8, 12]. In Finland, the proportion of people who thought that problem gambling is a serious issue and that gambling problems had increased rose between 2003 and 2011, and these differences were similar among both genders and different age groups [13]. Concurrently, the proportion of people without an opinion decreased, which may reflect an increased awareness and interest in gambling-related issues or normalization of gambling in general.

According to the earlier findings, women generally have more negative attitudes towards gambling than males [6, 8, 9, 14–16]. Young age, school qualifications, lower occupational status and higher level of income have been associated with positive attitudes towards gambling [6]. On the other hand, there is evidence that there are no associations between age and attitudes towards gambling [8].

As based on the theory of planned behaviour, attitudes towards behaviours, subjective norms, and perceived behavioral control influence individuals’ behavioural intentions and behaviour [17–22]. In earlier research, positive perceptions of gambling have been associated with greater intentions to gamble and increased gambling participation [6, 15, 23, 24]. The number of gambling activities involved during the past year or past week was associated with attitudes towards gambling [6]. Those who had at least five gambling activities during the past week had scores indicating positive attitudes towards gambling, but the number of past-year gambling activities was the strongest predictor. Furthermore, low onset age of gambling was associated with positive attitudes. Also, gambling frequency is positively associated with gambling behaviours, for example persistence of winning and borrowing money for gambling [25].

Non-gamblers and problem gamblers are less positive towards gambling than are non-problem and risk gamblers [6, 9, 15, 16]. Parental gambling has been associated with more positive attitudes towards gambling, but then the persons with either parents or other close relatives with gambling problems have generally less positive attitudes [6]. What, on the other hand, seemed to produce more positive attitudes towards gambling was good health; however, the health variables were weak predictors for attitudes while the impact of both smoking and alcohol consumption was moderate. On the other hand, health variables are associated with problem gambling [26–29]. The variables such as loneliness, smoking and risky alcohol drinking in particular were associated with problem gambling in a Finnish population study [30].

The aims of this study were to investigate the overall attitudes of Finnish population towards gambling, and to explore the association of socio-demographics, gambling behaviours, being a concerned significant other (CSO) of a problem gambler and also perceived health and lifestyle with attitudes towards gambling among the Finnish population. To our knowledge, our study is amongst the few conducted in the European context, and the first of its kind in Finland.

## Methods

### Participants

A random sample of 16 000 Finns were selected from the Finnish Population Register. Inclusion criteria were: 1) 15–74 years old, 2) mother language Finnish or Swedish, and 3) the residential area in mainland Finland. From that sample, 11 129 participants had a registered landline or mobile telephone number. In addition, 120 participants without a phone number were reached using a letter inquiring about their phone number and willingness to participate in the study. Finally, 11 249 Finns were approached using a telephone by a market research company Taloustutkimus Ltd. The survey was described to the respondents as “a gambling and health survey” [31]. Ultimately, 757 phone numbers were not valid, 1724 respondents could not be reached after maximum of 10 attempts, 4279 people refused to participate and five quitted during the interview.

### Setting

The gambling policy in Finland is based on a licensed system of monopoly with three operators. Veikkaus offers national lottery and betting games while Finland’s Slot Machine Association RAY offers slot machines, casino games and online games and Fintoto is responsible for the horse race betting. Of 15-74-year-old Finns, 78% had gambled during the past 12 months, and nearly half of the gamblers (46%) gambled on weekly basis [31]. The spending on gambling in Finland is one of the highest within the European Union [32]. The Finnish estimated cross-national problem gambling prevalence rate of 2.7% was measured using the South Oaks Gambling Screen (SOGS ≥ 3) [31]. Both Finnish cross-national and standardized problem gambling prevalence rates are equal with the rates in the United States, Canada, Australia and Sweden [2, 33].

### Procedures

The data were based on a cross-sectional population prevalence survey called: “The Finnish Gambling 2011” [30, 31]. All participants received written information about the upcoming study. The data were collected using computer-assisted telephone interview between 3rd of October 2011 and 14th of January 2012. Partial or complete interview was obtained from a total of 4484 participants. Accordingly, the response rate of the study was 40%. The data were weighted based on gender, age and region of residence (Northern, Eastern, Southern and Western Finland) [31]. The most underrepresented age groups included both males and females aged 15–19 and 25–34 while the most overrepresented age groups included 50–64 and 65–74 year-old-respondents. The weighted number of respondents was 4031. An approval to conduct the study was received from the Ethics committee of the National Institute of Health and Welfare. The ethical standards as laid down in the World Medical Association’s Declaration of Helsinki were followed [34].

*Attitudes towards gambling* were measured with the Attitude Towards Gambling Scale (ATGS-8) [6]. ATGS-8 is the 8-item version of the original 14-item version instrument developed for the British Gambling Prevalence Survey in 2007 [6]. ATGS-8 items were scored using a Likert scale: 1 = “strongly agree”, 2 = “agree”, 3 = “neither agree or disagree”, 4 = “disagree” and 5 = “strongly disagree”. Four items were reversely scored (Table 1: items 1, 4, 6, 7). The sum of eight items forms a total ATGS-8 score (range 8–40). A score of 24 is a midpoint and represents the overall neutral attitude towards gambling, while scores above 24 indicate an average attitude favourable to gambling and those below 24 unfavourable attitudes towards gambling [6, 8, 12].

**Table 1:** **Attitudes towards gambling items in Finland**

		Finland in 2011				Britain ^4^ in	
Item	N ^1^ 4031	Strongly agree or agree (%)	Neither agree or disagree (%)	Disagree or strongly disagree (%)	Mean ^2^ (SD)	2007 Mean ^2^ (SD)	2010 Mean ^2^ (SD)
**1. People should have the right to gamble whenever they want**	3987	53.0	6.4	40.6	3.17 (1.42)	3.38 (0.95)	3.57 (0.92)
**2. There are too many opportunities for gambling nowadays**	3953	73.0	9.5	17.5	2.09 (1.20)	2.08 (0.94)	2.08 (0.89)
**3. Gambling should be discouraged**	4001	91.9	3.8	4.3	1.43 (0.82)	2.55 (1.00)	2.69 (0.97)
**4. Most people who gamble do so sensibly** ^**3**^	3846	65.1	7.5	27.4	3.56 (1.28)	2.82 (0.97)	2.98 (0.97)
**5. Gambling is dangerous for family life**	3907	65.7	12.4	21.9	2.38 (1.16)	2.18 (0.96)	2.35 (0.94)
**6. On balance gambling is good for society** ^**3**^	3868	38.8	15.4	45.7	2.84 (1.28)	2.38 (0.88)	2.53 (0.88)
**7. Gambling livens up life** ^**3**^	3911	52.5	12.7	34.9	3.12 (1.26)	2.61 (0.98)	2.69 (0.94)
**8. It would be better if gambling was banned altogether**	3973	14.2	5.9	80.0	4.08 (1.16)	3.20 (1.05)	3.46 (0.97)
**Total score (sum of 8 items)**	3497	-	-	-	22.73 (5.56)	-	-

^1^Weighted based on gender, age and region of residence; ^2^Scale: 1 = “strongly agree”, 2 = “agree”, 3 = “neither agree or disagree”, 4 = “disagree”, 5 = “strongly disagree”; ^3^These items have been reversely scored so that all item means above 3.0 indicate an average attitude favourable to gambling and those below 3.0 unfavourable; ^4^The results from the British Gambling Prevalence Survey 2007 [12] and 2010 [8] are presented as reference information.

**Figure 1:**
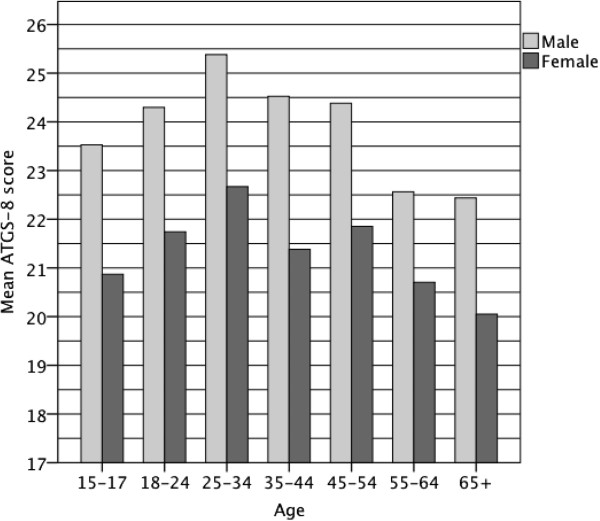
**Mean ATGS-8 score by gender and age.**

*Demographic characteristics* included respondent’s gender (male or female). Marital status was recoded into two categories: 1) married or cohabitating and 2) separated, divorced, widowed or single. In addition, three continuous variables were recoded. Age was recoded into 7 groups (Figure 1), education into two groups: 1) <12 years, or 2) ≥ 12 years, and net income into five groups: 1) 500€ or less, 2) 501-1000€, 3) 1001-1500€, 4) 1501-2000€ and 5) more than 2000 €. For the multivariate model, the net income categories 2–4 were combined.

**Figure 2:**
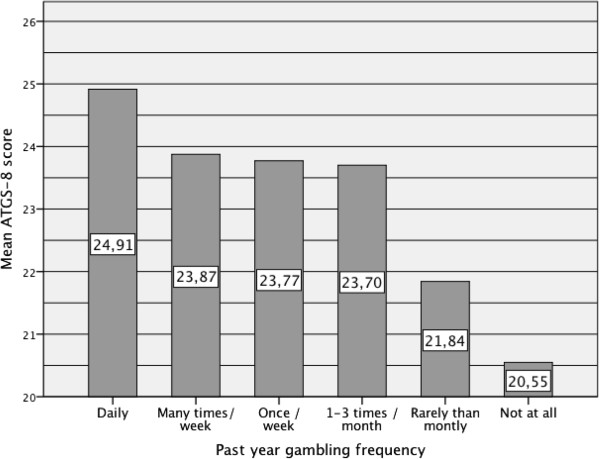
**Mean ATGS-8 score by past-year gambling frequency.**

**Figure 3:**
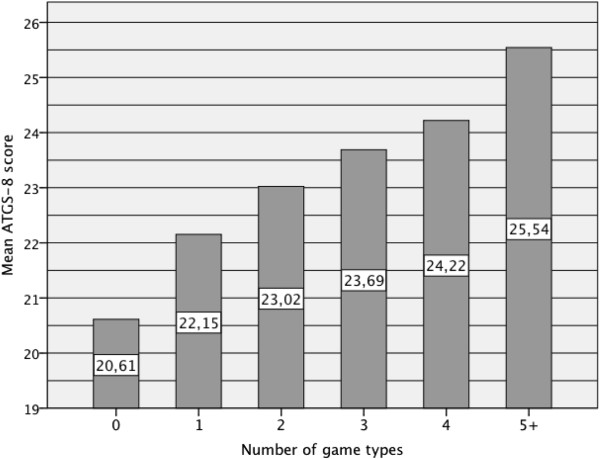
**Mean ATGS-8 score by the number of game types gambled during the past-year.**

*Gambling behaviours* included past-year gambling participation (any gambling; yes or no), past-year gambling frequency including six groups (Figure 2), number of game types gambled during the past year that was recoded into six groups (Figure 3) and onset age of gambling recoded into two groups: 1) <18 years and 2) ≥18 years. In addition, gambling severity was measured using a 12-month time frame with the SOGS originally developed by Lesieur and Blume [35, 36]. SOGS scores were: 1) 0–2 = non-problem gamblers and 2) ≥ 3 = problem or pathological gamblers, and non-gamblers were separated into the third group.

*CSOs* were identified with a question: “Has anyone of the following significant others of yours had problems with gambling? Then, seven choices including the father, the mother, a sister/brother, a grandparent, the spouse, own child/children and a close friend were named with three response options each (yes, no, do not know). A dichotomous (CSO/Non-CSO) variable was created to indicate whether the respondent had at least one significant other with gambling problems: response options “no” and “do not know” values were combined.

### Perceived health and lifestyle related variables

General health was inquired using a single question: “How is your general health at present?”. Self-rated health was recoded into two groups: 1) bad or somewhat bad and 2) average, good or somewhat good. Loneliness was inquired using a question: “Do you feel lonely?”, and it was recoded into: 1) sometimes, often or all the time and 2) never or very rarely. Smoking was inquired using a question: “Have you smoked during the past 12 months?”. Smoking was also recoded into two groups: 1) smoking daily or occasionally and 2) not at all. In addition, alcohol consumption was measured using a 3-item version of the Alcohol Use Disorders Identification Test (AUDIT-C) [37]. Total score for AUDIT-C was counted by summing up the points (range 0–3) for each item and using the cut-off points recommended for Finns to define risky drinking among males (≥6) and females (≥5) [38].

### Data analysis

The data were analysed using SPSS 21.0 software (SPSS, Inc., Chicago, IL, USA). Factor analysis was performed using maximum likelihood as an extraction method to test the structure of the factors of the ATGS-8 among the Finnish population. Factors were rotated with Varimax with Kaiser normalization. The results of the factor analysis supported the use of two factors, which is consistent with findings with the original 14-item instrument [8]. However, the first factor was substantially larger, since it accounted for 34% of variance while the other factor accounted for only 13% and looked like just a method factor contrasting positively and negatively worded items. Therefore, scoring the eight items as one scale was justified, as with the original instrument [6, 8]. Internal consistency reliability was assessed with Cronbach’s alpha coefficient. The Finnish version of the ATGS-8 reached the alpha value of 0.71 and the item-total correlations varied from 0.28 to 0.51. These figures were not very different compared with the British figures based on use of ATGS-8 where the alpha value was 0.76 and item-total correlations varied from 0.39-0.58 [12].

Descriptive statistics included frequencies, percentages, means, standards deviation (SD), median and quartiles. Statistical significance (*p*) was determined by one-way ANOVA and t-tests. The exact p-values are presented in the results to detect statistically significant differences (p ≤ 0.05). Multiple regression analysis (General Linear Model) was used to assess the effect (R square) of the demographic characteristics, gambling behaviour, being a CSO and health and lifestyle related variables. Both continuous, dichotomous (gender, education, CSOs, general health, loneliness, smoking) and categorical variables (gambling severity, net income) were included, and the dichotomous and categorical variables were treated as fixed factors. Marital status was omitted from the regression model since the four dummy variables needed did not contribute significantly in the model. All variables were included in the model simultaneously and treated as independent variables. Since gambling severity was measured using a validated instrument, it was considered as the most reliable measure to reflect gambling behaviour. Past-year gambling participation, past-year gambling frequency, number of game types gambled and onset age for gambling were omitted from the model. In the regression analysis, multicollinearity was weak.

## Results

### Respondents

A total of 4484 (2117 men, 2367 women) respondents aged 15–74 years (Mean 48.2; SD = 16.8 years) participated the study. Of the respondents, 41.3% had education less than 12 years and 48.3% were married or lived in a registered relationship. The past-year gambling prevalence was 77.9% and the number of game types gambled during the past year varied from 0 to 16 (Mean 2.2; SD = 2.02). The past-year problem gambling (SOGS ≥ 3) prevalence rate was 2.7%. Altogether, 19.3% of the respondents were identified as CSOs. In addition, 3.0% of the respondents perceived their general health bad or somewhat bad and 18.2% perceived themselves lonely (sometimes, often or all the time). 30.4% smoked daily or occasionally and 26.1% used alcohol at risky level (AUDIT-C ≥ 5 females, ≥6 males).

### Binary analyses

Total ATGS-8 scores (Range 8–40) were normally distributed. The overall sample mean score of 22.73 (Median 23.00) reflects unfavourable attitudes towards gambling (Table 1). The standard deviation of 5.56 indicates that there was plenty of individual variation around the mean value, with 50.0% of total scores lying between 19 and 27. Four items out of eight (items 2, 3, 5, 6) produced mean scores that suggest an average attitude unfavourable to gambling while the other four items produced means that suggest favourable attitudes (items 1, 4, 7, 8). The scores of items reflecting favourable attitudes (scores 3.12-4.08) were slightly closer to the midpoint of 3.00 than items reflecting unfavourable attitudes (scores 1.43-2.84).

The item that produced the most obvious expression of favourable attitudes towards gambling was: “It would be better if gambling was banned altogether” (item 8). Actually, 80.0% of respondents disagreed or strongly disagreed with this statement. Correspondingly, the item that produced the most obvious expression of unfavourable attitudes towards gambling was: “Gambling should be discouraged” (item 3). With this statement, 91.9% of the respondents agreed or strongly agreed. The item which received the largest percentage (15.4%) of neutral responses was: “On balance gambling is good for society”. Further analysis was performed to compare the scores between men and women. Mean scores for individual items indicated that men had statistically significantly (p ≤ 0.001) higher scores for each of the eight items.

Male gender and age between 18 and 54 were statistically significantly associated with total positive attitudes towards gambling (Table 2). Further analysis of the mean ATGS-8 scores was performed by gender and age (Figure 1). The mean scores among different age groups of women ranged from 20.05 to 22.67, which reflect women’s generally negative attitudes towards gambling. On the other hand, male respondents in all four age groups between 18 to 54 years had positive attitudes towards gambling with the scores ranging from 24.30 to 25.38, with under-18 s and over-54 s having on average negative attitudes.

**Table 2:** **Association between ATGS-8 and the correlates**

Variables	F/t	df	p	Positive attitude towards gambling ^1^ associated with:
**Socio-demographics**				
Gender (2 groups)	13.875	3495	p ≤ 0.001	Male gender
Age (7 groups)	16.530	6	p ≤ 0.001	Age groups between 18–54 years
Education in years (2 groups)	4.426	3495	p ≤ 0.001	12 years or more education
Marital status (4 groups)	5.434	3	p ≤ 0.001	Single status
Net income in Euro (5 groups)	7.968	4	p ≤ 0.001	Net wage > 2000 € or ≤ 500€ per month
**Gambling behaviours**				
Past-year gambling participation (2 groups)	12.151	3495	p ≤ 0.001	Any gambling
Past-year gambling frequency (6 groups)	46.643	5	p ≤ 0.001	High frequency
Number of game types, past-year (6 groups)	53.937	5	p ≤ 0.001	Large number of game types gambled
Onset age of gambling (2 groups)	9.331	3265	p ≤ 0.001	Onset age less than 18
Past-year gambling severity, SOGS^2^ (3 groups)	74.409	2	p ≤ 0.001	Non-problem gambler (score 0–2)
**CSO of a problem gambler (2 groups)**	7.448	3495	p ≤ 0.001	Non-CSO of the problem gambler
**Perceived health and lifestyle**				
Self-rated health (2 groups)	2.910	3489	p = 0.004	Good, somewhat good or average general health
Loneliness (2 groups)	2.026	3495	p = 0.043	Never or rarely lonely
Smoking (2 groups)	2.686	3495	p = 0.007	Smoking daily or occasionally
Alcohol consumption^3^ (2 groups)	4.689	3109	p ≤ 0.001	Risky alcohol consumption

^1^Total score for the Attitudes Towards Gambling Scale-8 (ATGS-8); ^2^SOGS, the South Oaks Gambling Screen, ^3^The Alcohol Use Disorders Identification Test (AUDIT-C), score for risky alcohol consumption ≥5 among women and ≥6 among men; CSO, concerned significant other of a problem gambler; Significance (p) is determined by one-way ANOVA (>2 groups) and *t*-test (2 groups); the data (n = 4484) were weighted based on gender, age and region of residence.

Single marital status was associated with the most positive attitudes towards gambling although the mean score of 23.26 remained on the negative side of the mid point of 24 (Table 2). Moreover, married respondents scored 22.42; cohabiting respondents 22.97 and divorced, separated or widowed 22.30. Respondents with at least 12 years of education scored higher compared to those with education shorter than 12 years (Mean 22.97 versus 22.03). The respondents in the centremost net wage group (1001–1500 €/month) had the most negative attitudes (Mean 21.87). The most positive attitudes were achieved among the lowest and highest net wage groups: the respondents with monthly net wage over 2000€ scored 23.05 and the respondents with the net wage of 500€ or less scored 22.70.

Generally, the association between gambling related variables and ATGS-8 scores were strong (Table 2). Past-year gambling participation was associated with higher ATGS-8 scores. However, the ATGS-8 scores above the mid point were reached only among daily gamblers with a mean score of 24.91 while the scores in the other groups varied from 20.55 to 23.87 (Figure 2). The greater amount of game types gambled on was associated with higher ATGS-8 scores (Figure 3). On average, respondents who gambled on less than four game types during the past-year had ATGS-8 scores (Mean 20.61-23.69) indicating negative attitudes, while the respondents who gambled on four or more game types had scores indicating positive attitudes (Mean 24.22-25.54). Among all respondents, non-problem gamblers (SOGS = 0-2) obtained the highest ATGS-8 scores (Mean 23.32). Onset age less than 18 years was associated with higher ATGS-8 scores compared with other respondents (Mean 23.71 versus 21.93).

CSOs of problem gamblers (Mean 21.33) had statistically significantly more negative attitudes towards gambling than other respondents (Mean 23.07). Good, somewhat good or average general health (Mean 22.78) as well as feeling never or rarely lonely (Mean 22.81) were associated with more positive attitudes towards gambling. In addition, smoking daily or occasionally (Mean 23.11) and, especially, risky alcohol consumption indicated positive attitudes (Mean 24.11).

### Multivariate model

Based on multiple regression analysis, the most significant variables associated with positive ATGS-8 scores were male gender, low age, net wage more than 2000€ and being a non-CSO (Table 3). Another statistically significant descriptive characteristic associated with positive ATGS-8 scores was 12 years or more education. Also low scores (SOGS = 0-1) on past-year gambling severity indicated significantly more positive attitude towards gambling than no past-year gambling or high scores on gambling severity (SOGS = 3+). High alcohol consumption was the only perceived wellbeing- and lifestyle-related variable which contributed significantly to positive ATGS-8 scores.

**Table 3:** **Multivariate models with the correlates and positive attitude towards gambling**

	Standardized beta coefficient	t value	p value
**Socio-demographics**			
Male gender	2.049	9.972	<0.001
Female gender	a	a	a
Age in years	-0.044	-6.229	<0.001
12 years or more education	0.787	3.408	<0.001
Less than 12 years education	a	a	a
Net income 500€ or less	-0.413	-1.941	0.052
Net income 501-2000€	-0.864	-2.242	0.025
Net income >2000€	a	a	a
**Gambling behaviours**			
No past-year gambling	-2.066	-8.441	<0.001
Problem or pathological gambler, SOGS = 3+	-1.875	-3.291	<0.001
Non-problem gambler, SOGS = 0-2	a	a	a
**Non-CSO of problem gambler**	1.757	7.540	<0.001
CSO of problem gambler	a	a	a
**Perceived health and lifestyle**			
Good, somewhat good or average general health	1.136	1.929	0.054
Bad or somewhat bad health	a	a	a
Never or rarely lonely	0.025	0.104	0.917
Sometimes, often or all the time lonely	a	a	a
Daily or occasional smoking	-0.253	-1.181	0.238
Do not smoke at all	a	a	a
Risky alcohol consumption, AUDIT-C score	0.192	3.901	<0.001

Multiple Regression Analysis; Summary statistics: R^2^ = 0.125, adjusted R^2^ = 0.121, F = 37.232, df = 12, p < 0.001; ATGS-8, the Attitudes Towards Gambling Scale-8; AUDIT-C, The Alcohol Use Disorders Identification Test; SOGS, the South Oaks Gambling Screen; CSO, concerned significant other of a problem gambler; a, reference group.

## Discussion

### Overall attitudes towards gambling

Overall attitudes towards gambling among Finns were generally negative. This finding is consistent with other studies on gambling attitudes that were conducted in countries where legal gambling opportunities are widely available [6–9, 39]. Comparing both the eight individual items measuring the attitudes (Table 1) and the total ATGS-8 scores, the perceptions were mostly more favourable towards gambling in Finland than in Britain [6, 8, 12]. British respondents had more favourable attitudes based on only two items: agreeing that “people should have the right to gamble whenever they want” and disagreeing that “gambling should be discouraged”.

### Factors associated with attitudes towards gambling

Both higher total scores and higher mean scores for individual items reflected consistently more positive attitudes towards gambling among men over women. This finding is consistent with the results of the British prevalence studies [6, 8, 12]. The latter also found unfavourable attitudes towards gambling among all age groups of both women and men [8]. Contrary to their results, our results imply that in general Finnish males aged 18–54 years had scores indicating positive attitudes towards gambling.

The relationship between gender and gambling attitudes also reflects gambling behaviour since men and younger individuals typically gamble more and have a higher risk for gambling problems [2, 33, 40]. The legal age for gambling in Finland was raised from 15 years to 18 years in 2011 to protect the under-aged from potential gambling-related harms. Despite the increase in age limit for gambling in Finland, past-year gambling participation of 15-64-year-old Finns has increased significantly between 2007 and 2011; however, this change was mainly explained by occasional gambling (less seldom than monthly) of women aged 25–34 and 50–64 years [13]. In Britain, the biggest change towards positive attitudes was seen among the 55-year-olds or older population between 2007 and 2010 [12].

In the binary analysis, an intriguing finding was that both lower and higher net wage groups held more positive attitudes towards gambling compared with other respondents. The finding for the lower net wage group may also be consistent with the findings of studies on problem gamblers: low income and unemployment are associated with gambling problems [2, 33]. One explanation is that perhaps lower socio-economic status puts individuals in a risk-prone position, making them more vulnerable to gambling-related cognitive erroneous beliefs [41].

According to our binary analysis, high gambling frequency and high number of game types gambled were associated with favourable attitudes towards gambling. This is in line with the theory of planned behaviour [17–19] and previous research [6, 20–22]. However, our results are not fully comparable with TBP because we used only a limited number of TBP elements. We also found a significant association between early onset age of gambling and positive attitudes towards gambling. In the multivariate model, we found an association between low SOGS scores and positive attitudes, indicating that those gamblers who did not have problems with gambling held more positive attitudes towards gambling. Moreover, being a CSO was one of the most important factors associated with negative attitudes towards gambling, which is in line with the British study [42]. One explanation for these relationships is that who have experienced problems, either themselves or in the family or friends, may have developed negative attitudes as a results.

Finally, high alcohol consumption was the only health and lifestyle related variable significantly associated with positive attitudes towards gambling. These findings are in line with the results of the British Gambling Prevalence Survey in 2010 [6], but not as assumed based on the findings among problem gamblers [26–30]. Risky alcohol consumption, however, is associated with both problem and pathological gambling in Finland [30, 43] and internationally [33, 40, 44].

### The impact of the contextual factors

Contextual determinants, for example availability, accessibility and exposure to advertisements, are thought to impact attitudes towards gambling [45]. In Finland, gambling opportunities and incentives to gamble are very visible. For example, the amount of slot machines in Finland is one of the highest in Europe (approximately 20,000 machines) and different types of games are widely available in grocery stores, kiosks, restaurants and gas stations. Finns can also gamble via the internet - not only the internet gambling offered by the national gambling operators but also by international gambling operators. Advertisements of gambling activities are also very visible in Finland and often linked with hope, glamour, lust and joy. As part of the profits of the Finnish monopoly system are used to promote public good (for example to support Finnish health and welfare, science, arts, youth work and sports), gambling in Finland is often advertised using phrases such as “Gamble for public health” (“Pelaa kansanterveydelle!”). This can partly explain why the Finns have more favourable attitudes towards gambling than, for example, British respondents [6, 8, 12]. A high level of gambling advertisements in a society is likely to lead to the normalisation of gambling and perception of gambling as an acceptable and harmless activity [46].

Acknowledgement of these contextual determinants affecting attitudes towards gambling is important when planning preventive measures and risk reduction models for gambling. To reduce gambling-related harms, clear and appropriate information about gambling products should be offered, as stated in the Reno Model [47] and recommended by the European Commission [48].

### Limitations

International comparison of the population based gambling studies indicate that the response rate of this population study was below the international average [2]. A power analysis was not calculated when determining the sample size. To improve the sample representativeness, the data were weighted based on age, gender and region of residence [31]. The Finnish version of the ATGS-8 was used for the first time. The Finnish translation was created using a qualified translator, collaboration with an expert panel and a pilot test (N = 30). Two of the authors of the 2007 and 2010 British Gambling Prevalence Surveys [8, 12], who developed the original English version of ATGS-8, checked the back-translation. Based on their feedback, the future Finnish translation of the term “sensibly” in the item 4 could be improved (the back-translation suggested that the current Finnish word used might be closer in meaning to the English phrase ‘in moderation’). The interpretation of the results related to being a CSO is limited by the fact that the gambling problems of the respondents’ significant others were based assessed with a single item-measure. Therefore, the concern mainly reflects the potential existence of the gambling problems of the significant others without any evaluation of the amount or type of concern. Finally, our findings may not be generalizable to other cultural contexts where, for example, different gambling regulatory frameworks are present.

## Conclusions

Despite generally negative attitudes towards gambling overall, male gender, younger age, high net income and risky consumption of alcohol were associated with more favourable attitudes towards gambling. These relationships reflect associations with actual gambling behaviour, since men, younger individuals and riskier alcohol consumers typically gamble more and have a higher risk for gambling problems, and in the present study more positive attitudes were associated with frequency and extent of gambling. Experiencing negative consequences of gambling caused by either one’s own or a significant other’s gambling seems to result in more unfavourable attitudes. The Finnish version of ATGS-8 can be used in the future to follow the possible changes in attitudes of the Finns and furthermore to compare the possible changes internationally. Monitoring population attitudes towards gambling is relevant to understanding, i.e. the impact of gambling policy and the changes in it. All in all, more studies are needed to study further the impact of contextual factors on attitudes.
